# Where to start? The Irish Emergency Department Antimicrobial Discharge (EDAD) study: a multicentre, prospective cohort analysis

**DOI:** 10.1093/jacamr/dlae038

**Published:** 2024-03-12

**Authors:** Aisling Rafferty, Alida Fe Talento, Richard Drew, Patrick Fitzpatrick, Kara Tedford, Michael Barrett, Husnain Mahomed, Sabrina O’Regan, Louise Delany, Síle O’Connor, Agne Buseckyte, Andrei Brovchin, Elhaytham Hassan, Anna Marzec, Donna Martin, Clare Greene, John Marriott, Robert Cunney

**Affiliations:** Department of Pharmacy, Children’s Health Ireland at Temple Street, Dublin, Ireland; School of Pharmacy, Institute of Clinical Sciences, University of Birmingham, Birmingham, UK; Department of Microbiology, Children’s Health Ireland at Temple Street, Dublin, Ireland; Department of Microbiology, Royal College of Surgeons, Dublin, Ireland; Department of Microbiology, Trinity College Dublin, Dublin, Ireland; Department of Microbiology, Children’s Health Ireland at Temple Street, Dublin, Ireland; Clinical Innovation Unit, Rotunda Hospital, Dublin, Ireland; Irish Meningitis and Sepsis Reference Laboratory, Dublin, Ireland; Emergency Department, Children’s Health Ireland at Temple Street, Dublin, Ireland; Department of General Paediatrics, Royal College of Surgeons, Dublin, Ireland; Department of Pharmacy, Children’s Health Ireland at Crumlin, Dublin, Ireland; Emergency Department, Children’s Health Ireland at Crumlin, Dublin, Ireland; Emergency Department, Children’s Health Ireland at Crumlin, Dublin, Ireland; Department of Pharmacy, Portiuncula University Hospital, Galway, Ireland; Department of Pharmacy, National Maternity Hospital, Dublin, Ireland; Department of Pharmacy, University Hospital Kerry, Kerry, Ireland; Emergency Department, University Hospital Kerry, Kerry, Ireland; Emergency Department, University Hospital Kerry, Kerry, Ireland; Emergency Department, University Hospital Kerry, Kerry, Ireland; Department of Pharmacy, Our Lady’s Hospital Navan, Meath, Ireland; Department of Pharmacy, Cavan General Hospital, Cavan, Ireland; Department of Pharmacy, Midland Regional Hospital, Tullamore, Offaly, Ireland; School of Pharmacy, Institute of Clinical Sciences, University of Birmingham, Birmingham, UK; Department of Microbiology, Children’s Health Ireland at Temple Street, Dublin, Ireland; Department of Microbiology, Royal College of Surgeons, Dublin, Ireland; Irish Meningitis and Sepsis Reference Laboratory, Dublin, Ireland

## Abstract

**Objectives:**

To determine the percentage of patients across Ireland who are discharged from the Emergency Department (ED) with an antimicrobial prescription, the indication, classification of infections, and guideline compliance. To identify potential areas for antimicrobial stewardship (AMS) interventions in the ED.

**Patients and methods:**

A multicentre, prospective cohort analysis study in EDs across eight hospitals in Ireland. At each site, patients aged 1 month and older who presented to the ED and were discharged directly from the ED were included. A random selection of records of patients discharged from the ED were reviewed until a minimum of 30 records with an infection diagnosis resulting in an antibiotic prescription were obtained per hospital. The number of patient discharges with no antibiotic prescriptions were included to calculate the denominator. The indication, infection classification and guideline compliance data were collected on the 30 prescriptions in the participating hospitals.

**Results:**

A total of 2619 patient records were reviewed. Of these, 249 (9.5%) patients were discharged with antimicrobial prescriptions from the ED. Most (158; 63%) were classified as probable bacterial infection, 21 (8%) as probable viral, and 18 (7%) had no documented evidence of infection. Three indications accounted for 73% of antimicrobial prescriptions: skin/soft tissue infection; ear, nose and throat infection; and urinary tract infection. Overall guideline compliance was 64%.

**Conclusions:**

Several areas for AMS interventions to optimize antimicrobial prescribing in the ED were identified, including targeted local and national guideline reviews, delayed prescribing, improved point-of-care testing and prescriber and patient education.

## Introduction

Antimicrobial resistance (AMR) is in the top five of world health risks.^1,2^ AMR results in increased length of stay, mortality rates and cost burden on patients, society and our healthcare systems.^1,2^ Reducing inappropriate use of antimicrobials has been described as our most important intervention to prevent AMR.^3,4^ Antimicrobial stewardship (AMS) programmes are an internationally recommended method of promoting appropriate antimicrobial prescribing and thereby combating AMR.^5^ AMS programmes have the additional benefit of reducing the duration of therapy, preventing treatment failure, reducing side effects associated with antimicrobials and reducing costs.^6–8^

The Emergency Department (ED) is the perfect setting for an AMS intervention as it is the interface of hospital and community in all healthcare systems.^9^ Stewardship programmes rarely target this interface for their initiatives,^7^ instead tailoring interventions towards doctors in training and in-patient prescribing practices.^10^ The ED presents challenges to effective AMS interventions, compared with inpatient settings. For example, there are more time constraints with higher patient turnover that may influence prescribing decisions.^11^ Studies from the USA have shown that at least 15% of patients who attend the ED go home with a discharge prescription for an antibiotic^12^ and it is estimated that up to 10 million prescriptions for antimicrobials are written in EDs yearly.^13^ A systematic review carried out by Losier *et al*. aimed to inform best practice AMS interventions in the ED, thus improving patient outcomes and reducing the negative consequences of antimicrobial prescribing. Losier and colleagues concluded that while AMS in the ED is beneficial to patients’ treatment, the optimal combination of interventions is unclear and therefore needs more research.^7^

AMS requires customization so that it responds to local needs, behaviours and resources.^14^ Literature on antimicrobial prescribing at the time of discharge from the ED is lacking outside the USA and Canada. To determine the optimal combination of targeted AMS interventions, thus informing best practice, baseline data on antimicrobial prescribing need to be determined. The present investigation is an Irish, multicentre, prospective cohort analysis study and aimed to identify areas to target ED AMS interventions. The primary objective was to determine the percentage of patients across Ireland who are discharged from the ED with an antimicrobial prescription. The secondary objective was to determine the classification of infections, indications resulting in an antimicrobial prescription and guideline compliance.

## Methods

This prospective cohort study was reviewed and approved by the Children’s Health Ireland (CHI) Research and Ethics Committee (Reference no: CA21-09-02).

### Expression of interest

Expression of interest was circulated to the Irish Antimicrobial Pharmacist Group (IAPG) to participate in the study. The group consists of antimicrobial specialist pharmacists working in hospitals across Ireland. From the 45 members, 14 expressed interest and 8 enrolled in the study. Individuals at participating sites sought approval from their local committees to take part in the study. After enrolment the study protocol and data collection tool were shared with each site. A meeting was held with all centres and the study protocol was discussed and feedback received. Amendments were made to the data collection tool and protocol based on expert opinions of the antimicrobial pharmacist, consultant microbiologist and ED consultants who were responsible for the data collection. The protocol amendments took into account infrastructural differences between the hospitals. After agreement of the protocol design, education on data collection was carried out to ensure consistency of the data.

### Setting

**Figure 1.**
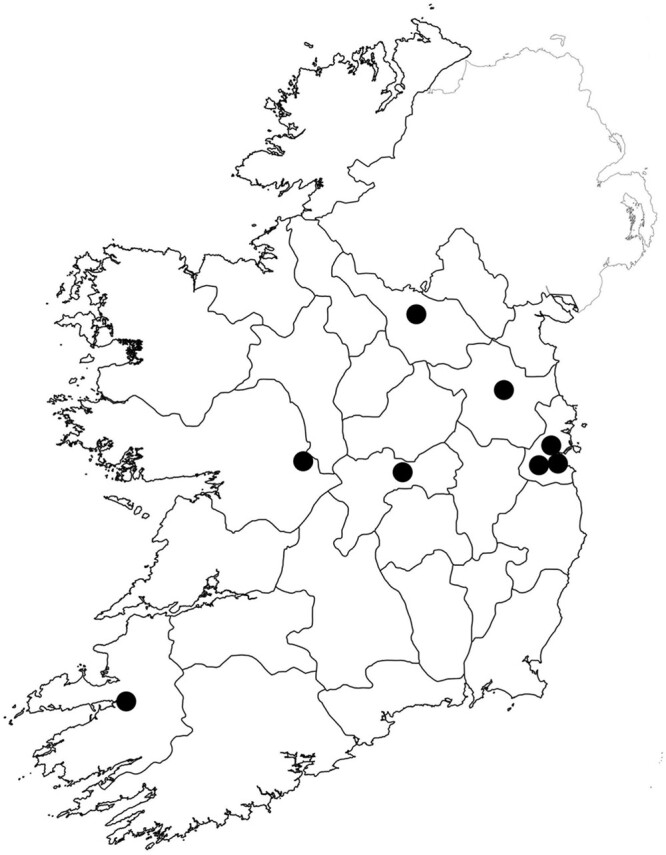
Graphical representation of the geographical location of the eight participating hospitals in the Republic of Ireland: Children’s Health Ireland at Temple Street; Children’s Health Ireland at Crumlin; National Maternity Hospital; Cavan General Hospital; Our Lady’s Hospital Navan; University Hospital Kerry; Midland Regional Hospital Tullamore; and Portiuncula University Hospital. Image as per copyright©worldmapblank.com.

The eight hospitals that enrolled included two tertiary and quaternary speciality paediatric centres, one tertiary-level speciality maternity hospital and five Model 3 hospitals (these admit undifferentiated acute medical patients, have an Acute Medical Assessment Unit, an ED on site and a category 1 or 2 ICU).^15,16^ The geographical location of the hospitals involved can be seen in Figure 1. In Dublin, specialties such as maternity and paediatric hospitals are separate locations to that of the main adult centres. To give an indication of the antimicrobial discharge rates across Ireland these centres have been included to ensure specialist populations are considered.

### Population

Patients aged 1 month and older were included in the study if they presented and were discharged directly from the ED, including those who were discharged to a long-term care facility.

#### Exclusion criteria

Patients were excluded if they were less than 1 month old, if they were admitted as an inpatient or in a short stay unit, or if they were transferred to another acute care setting (e.g. another hospital ED). Long-term prophylactic antimicrobials and topical antimicrobial agents were not included, as we wanted to focus on prescribing of systemic agents prescribed for acute infections.

### Design and data collection

Each hospital provided details on availability of local empirical antimicrobial guidelines and their availability of point-of-care testing (POCT). Prospective data were collected from patients discharged directly from the ED during March to September 2022. The data collection period was targeted to be outside of the viral winter period to remove seasonality as a contributing factor to prescribing practices. Patient records were reviewed using a convenience sampling method (i.e. not necessarily consecutive discharges), meaning discharges may have occurred on any day of the week or during any time of the day or night. Using convenience sampling rather than consecutive chart selection removes the bias around prescribing practices or behaviours that may be associated with one team, time of day, or day of the week.

To calculate sample size requirements, a sample size calculator was used based on the Ireland population of 5.033 million. The sample size set the minimum requirements for the number of overall charts required for review to determine the percentage discharged with an antimicrobial prescription.^17^ The percentage of patients discharged with an antimicrobial prescription were calculated per hospital, an overall average and an overall median. Patients’ discharges were reviewed until a minimum of 30 records with an infectious diagnosis resulting in an antibiotic prescription were obtained. The discharges with no antibiotic prescriptions had no further data on demographics or discharge prescription details collected but were included in the denominator.

Further data were collected on the minimum of 30 discharge antimicrobial prescriptions at each site. The datapoints collected were patient demographics, allergy status documentation, laboratory evidence of infection [e.g. raised WBC count (WCC), raised C-reactive protein (CRP), viral or bacterial PCR diagnostics, point-of-care Group A streptococcal antigen detection (‘rapid strep test’), urinalysis or culture results], the indication, if a provisional or delayed prescription was given, the antibiotic prescribed and duration of treatment. Each indication was classified as per the PERFORM study:^18^ definite bacterial; probable bacterial; definite viral; probable viral; definite fungal; probable fungal; or no evidence of infection. The definitions of definite infections were based on clinical signs or symptoms and laboratory, point-of-care or radiological findings consistent with infection. Probable infection was defined as clinical signs or symptoms, radiological findings or raised inflammatory markers suggestive of infection. The definition of no evidence of infection was absence of laboratory, point-of-care, clinical signs or symptoms, radiological findings, or raised inflammatory markers suggestive of an infection, or no documentation of an infection in the patient’s records.^18^

Guideline compliance was recorded by the data collector. A prescription was compliant if the choice of antibiotic and duration was as per the local hospital guideline or had been advised on consultation with a microbiology/infectious diseases physician. If the antibiotic or duration of treatment was not documented, the prescription was deemed compliance unknown. If there was no appropriate guideline available, such as guidelines on penicillin allergy, it was classified as ‘no guideline available’.

### Data analysis

Each hospital collected their local data and returned them for data analyses. Data were irrevocably anonymized during data collection. Each hospital’s name was replaced with a pseudonym during data analysis. Indications were grouped as per national Health Service Executive (HSE) guidelines.^19^ Cluster effect analysis was carried out per hospital using Excel (Microsoft Corporation, 2013). Specific antibiotic prescribing per indication and duration analysis was conducted if five or more patients were treated for the specific indication (i.e. ≥5 prescribing episodes). Further analysis was carried out for subclasses of indications if ≥5 prescribing episodes occurred, e.g. five or more patients were treated for a skin/soft tissue infection (SSTI) associated with a wound; wound SSTIs excluded animal or human bites. The indication was prophylaxis of SSTI, referring to an antimicrobial given to prevent an SSTI from occurring after an injury; 95% CIs were calculated for durations per indication.

## Results

**Figure 2.**
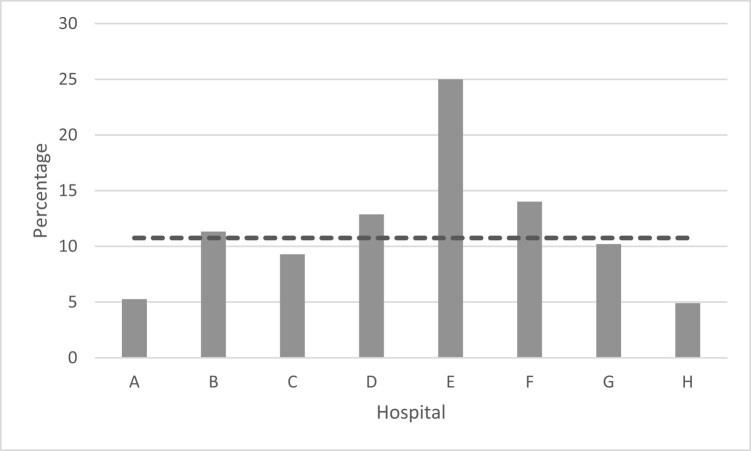
Percentage of patients discharged with an antimicrobial prescription across the eight EDs. The median (dotted line) was 11%.

A sample size of greater then 385 charts gives an estimated discharge rate of antimicrobial prescriptions with a 99% CI.^17^ A total of 2619 patient records were reviewed. Of these, 249 (9.5%) patients were discharged with antimicrobial prescriptions from the ED. The median proportion across the eight participating hospitals was 11% (range 5%–25%; Figure 2). An average of 9.5% of patients are discharged with an antimicrobial prescription from the EDs across Ireland with a 99% CI. Details of patient demographics per hospital are in Table 1. Of the 249 patients, allergy status was documented in 246 (99%), of which 18 (7%) had an antibiotic allergy where 15 (83%; 6% of total) had a documented penicillin allergy.

**Table 1.** Summary of patient demographics seen across eight EDs in Ireland who were discharged with an antimicrobial discharge prescription

Hospital	Gender female, % (*n*)	Age (years), median (range)	Weight if age < 16 years (kg), median (range)
A	66 (19)	4 (0.4–14)	20.3 (7.5–60)
B	67 (20)	3 (1.2–15)	16.65 (9.1–79.3)
C	50 (15)	47.5 (1–88)	74.1 (10–76)
D	47 (14)	47.5 (17–90)	NA
E	55 (23)	46 (<1–97)	22 (10.9–39.8)
F	57 (17)	39.5 (0.6–90)	19.3 (13.5–26)
G	100 (30)	33 (19–44)	NA
H	54 (15)	53 (11–90)	48 (48)
Across all EDs	61 (153)	31.5 (0.41–97)	20.4 (7.5–79.3)

NA, not applicable.

### Guideline availability, POCT and rapid diagnostics

All sites had local antimicrobial prescribing guidelines, and all sites had guidelines available on a mobile application platform. POCT was available in five (62.5%) hospitals. Rapid diagnostics for COVID-19 in the ED were available in 62.5% (*n* = 5), influenza in 50% (*n* = 4) and full viral panel in 12.5% (*n* = 1). One hospital documented having rapid Group A streptococcal antigen detection from throat swabs. No hospitals documented further bacterial rapid diagnostic tests being available.

### Classification of infection

Nighty-one patients (37%) had laboratory evidence of infection, of which 86 (94%) were bacterial, 2 (2%) viral and 1 (1%) fungal. The majority of cases prescribed antibiotics were classified as probable bacterial infection (158; 63%). Definite bacterial infections were confirmed in 48 (19%) cases. Only three (1%) cases had probable fungal infections, 21 (8%) were probable viral, and 18 (7%) had no documented evidence of infection.

### Indication

**Figure 3.**
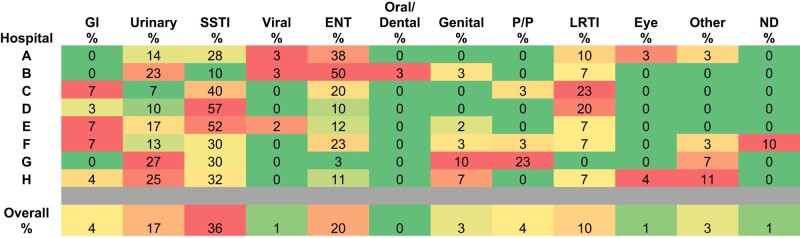
Heat map demonstrating a cluster effect analysis of the percentage of infectious indications that resulted in antibiotic prescribing from the ED from each hospital and in total. The heat map can be read per row (indications) or per column (hospitals) separately or assessed in its entirety. Red indicates high prescribing rates between the comparators, either rows (indications) or columns (hospitals), yellow/orange indicates medium prescribing rates and green indicates the lowest rates of prescribing when compared with other hospitals or indications. GI, gastrointestinal; LRTI, lower respiratory tract infection; P/P, pregnancy/postpartum; ND, not documented.

A heat map demonstrating the percentage of presenting indications that resulted in antibiotic prescription across the eight hospitals can be seen in Figure 3. Three indications accounted for 73% of antimicrobial prescriptions, namely SSTI, ear, nose and throat (ENT) infections, and urinary tract infections (UTIs). Within the 89 patients with SSTI, 14 (16%) were prescribed antimicrobials as prophylaxis and 19% (*n* = 17) had SSTI associated with a wound. Of the 42 patients with a UTI only 1 was documented as having pyelonephritis.

### Guideline compliance and antibiotic prescribing

**Figure 4.**
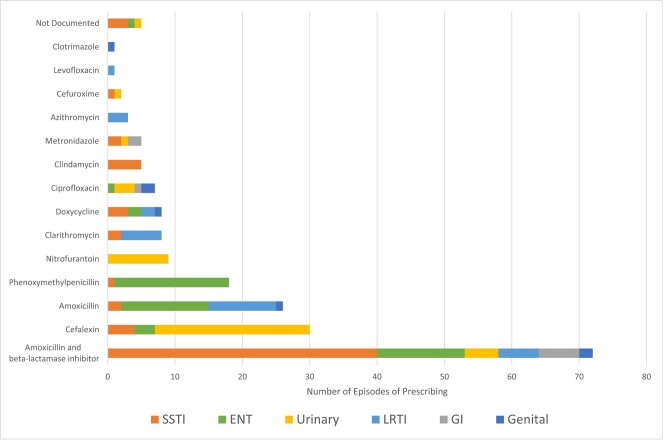
The most frequently prescribed antibiotics by indication for five or more prescribing episodes of the 249 discharge prescriptions. LRTI, lower respiratory tract infection; GI, gastrointestinal.

One hundred and sixty (64%) prescriptions were based on local hospital guidelines, 72 (29%) were non-compliant with local guidelines, 8 (3%) were associated with no available guidelines and in 9 (4%) cases guideline compliance was unknown. Delayed or provisional prescriptions were given in 5 (2%) of cases, all of which were classified as probable viral and upper respiratory infection. Figure 4 displays the prevalence of antibiotic prescribing per indication. Furthermore, co-amoxiclav was prescribed for SSTI associated with a wound in 17 (100% of cases) and to 9 (53% of cases) for SSTI prophylaxis.

### Duration

The duration range, median and 95% CI for indications with ≥5 prescribing episodes can be seen in Table 2.

**Table 2.** The duration for each indication classification and additional subclasses of indications diagnosed in five or more patients, of the 249 discharge prescriptions, e.g. SSTI associated with an abscess or wound, ENT subclass ‘Otitis media’

Indication	*n*	Median	Range	95% CI
GI	9	7	5–7	(5.1–6.89)
Urinary	42	5	3–7	(4.46–5.7)
SSTI	89	7	5–14	(6.16–6.85)
Cellulitis	39	7	5–14	(6.32–6.59)
Abscess	7	7	7–10	(6.21–8.78)
SSTI—wound	17	7	5–7	(5.58–6.67)
SSTI—prophylaxis	14	5	3–7	(4.23–5.76)
ENT	51	7	3–14	(6.55–7.9)
Otitis media	9	8.5	10–5	(5.1–9.75)
Tonsillitis	26	7	3–10	(7.05–8.94)
URTI	5	7	5–7	(4.73–7.77)
Genital	8	10	3–14	(0.45–11.6)
LRTI	25	5	3–7	(5.2–6.35)
CAP	21	5	3–7	(5.07–6.4)
Pregnancy/postpartum	9	7	5–7	(5.15–7.13)

Indications with fewer than five prescriptions were not included. SSTI—wound excludes animal or human bites. GI, gastrointestinal; LRTI, lower respiratory tract infection; URTI, upper respiratory tract infection; CAP, community-acquired pneumonia.

## Discussion

To our knowledge this is the first multicentre prospective study that investigated the antimicrobial prescribing practices at the time of discharge from the EDs across Ireland. The heterogeneity of this study reflects the real-life discharge practices in the participating EDs. We found that 9.5% of patients discharged from the ED were prescribed an antimicrobial agent. The USA estimates 15% of patients are discharged from the ED with an antimicrobial prescription.^12^ Using this as a comparator we are achieving lower prescribing rates. A rate of 9.5% can now be used as a benchmark to gauge the impact of AMS interventions within the ED and provide hospitals nationally with a comparator for their rates of prescribing. The average age seen was 31.5 years (range 0.4 to 97) and 61% were female. Table 1 demonstrates the breadth of patient demographics that were seen.

### Guidelines

Nationally and locally, AMS programmes could focus on SSTI, ENT and UTI indications as a priority in the ED as they were responsible for 73% of prescriptions in our study. Reviewing national and international guidelines for SSTI, in particular prophylaxis and wound-related infection, the national HSE prescribing guidelines and the NICE guidelines for adults and children reference SSTI prophylaxis in patients with recurrent cellulitis and little is stated regarding wound-related cellulitis.^19,20^ Our findings highlight the need to expand the national and local SSTI guidelines to include prevention of infection after an injury: when to or when not to prescribe antibiotics, duration, and additionally add or expand on guidance for infected wound-associated cellulitis. Future studies are warranted to examine the reason why guideline non-compliance rates are as high as 29%, as another potential AMS intervention target.

### POCT and delayed prescriptions

The analysis of POCT in combination with infection classification highlighted areas of opportunities for AMS interventions. The potential for expansion of POCT testing in ED could reduce the ED length of stay and direct care if integrated into the ED existing processes.^21,22^ Using POCT could shift the percentage of probable cases to definite bacterial and/or definite viral diagnosis. For the probable viral cases (8%) and no evidence of infection cases (7%), POCT and delayed prescriptions could play a role in reducing antimicrobial consumption. Only 2% of patients had a delayed or provisional prescription given. Studies have shown that provisional prescriptions, particularly when given with a patient information leaflet, can greatly reduce antimicrobial consumption by the patient. In one study, 20%–46% of patients consumed the prescribed antimicrobial during the course of their illness if they received a delayed prescription, while patients who received immediate prescriptions had a consumption rate of 96%–99%.^23^ The targeting of ‘no evidence of infection’ and ‘probable viral’ infections has the potential to reduce antimicrobial prescribing at discharge by 15%.

### Duration

Otitis media is more common in paediatrics and most cases resolve spontaneously. The duration of treatment for otitis media ranged from 5 to 10 days. National guidelines recommend 5 days, which can be extended to 7 days if severe.^19^ This is an example of an AMS intervention targeting duration to reduce antimicrobial use and exposure.

### Primary and urgent care

A national AMS programme focused on inappropriate prescribing in the community using a traffic-light system for appropriate antimicrobials by indication.^24^ One of the main focuses of improvement was the reduction of co-amoxiclav prescribing from 33% to 10% post intervention.^24^ Data from this study informed the downgrading of indications for co-amoxiclav in the national UTI guideline.^24^ This work is reflected in the present study and can be seen in Figure 4, where the antibiotics prescribed for the majority of UTIs were cefalexin and nitrofurantoin, which is compliant with the national guidelines.^19^ This indication approach could be used in the ED departments to optimize antimicrobial prescribing.

Nationally the healthcare system is overburdened, where GP and ED waiting times are higher than national targets.^25^ It could be argued that some of the patients discharged from the ED could be managed as successfully in primary care or urgent care. For example, of the patients presenting with UTI, only one had pyelonephritis. We noted a wide range of durations of antibiotics used, indicating that not all infections seen were classified as severe, which usually require longer durations of therapy. Further work could be carried out on national guidelines to include the slightly more complex patients or look at pathways outside of the ED that can facilitate these patients such as an urgent care centre. An urgent care unit in proximity to a hospital has been shown to reduce waiting times in the ED.^26^ Furthermore, interventions such as the one carried out by O’Connor and colleagues^24^ could be used in this setting in combination with POCT and delayed prescribing to target these patients.

## Limitations

Most participating hospitals used either a combination of electronic and paper-based patient records, or solely paper-based systems. This may have resulted in some undocumented data owing to filing, lack of documentation or poor data transfer on carbon copies of prescription pads. Recruitment of hospitals was carried out through the IAPG network. This may have introduced bias as those hospitals with an AMS pharmacist may have established stewardship programmes in place, compared with those hospitals without. While 2619 patient records were reviewed to determine the proportion prescribed antimicrobials, detailed data analysis was carried out on 249, which is a small sample size when the data are broken down into different indications. The powering of the study was based on determining the rate of prescribing. The secondary aim power was not reached as the number of charts reviewed for data collection was based on the original 14 centres who expressed interest. Due to a national pharmacist staffing crisis, there were six centres that subsequentially could not take part, which led to the secondary aim being underpowered. However, as the patient cohort and demographics were widely spread across Ireland with different specialities and district hospitals, the data while small in some instances, can be extrapolated to represent the wider population. The impact of the winter viral season was not assessed. The removal of the winter viral season was intentional to remove the bias of the winter surge of infection. This should be investigated in future studies. The data provided by this study can be used as a comparator for future winter season investigations to assess if the rate of antimicrobial prescriptions increases during the winter season and if there is a shift in the indications more likely to result in antimicrobial prescribing.

### Conclusions

Of the patients discharged from the ED, 9.5% of them left with an antimicrobial prescription. We have identified several areas for targeted AMS interventions to optimize antimicrobial prescribing in the ED. These include availability of POCT, prescriber and patient education regarding antimicrobial prescribing for patients with viral infections, delayed prescriptions, review of guideline non-compliance, local and national guidelines with a focus on duration of therapy and the role of primary or urgent care in AMS.
